# Predictive value of cystatin C and neutrophil gelatinase-associated lipocalin in contrast-induced nephropathy: A meta-analysis

**DOI:** 10.1371/journal.pone.0230934

**Published:** 2020-04-02

**Authors:** Yi He, Yunzhen Deng, Kaiting Zhuang, Siyao Li, Jing Xi, Junxiang Chen

**Affiliations:** Department of Nephrology, Hunan Key Laboratory of Kidney Disease and Blood Purification, The Second Xiangya Hospital of Central South University, Changsha, Hunan, China; University of Sao Paulo Medical School, BRAZIL

## Abstract

**Background:**

There are still limited studies comprehensively examining the diagnostic performance of neutrophil gelatinase-associated lipocalin (NGAL) and cystatin C in contrast-induced nephropathy (CIN). The study aimed to investigate and compare the predictive value of NGAL and cystatin C in the early diagnosis of CIN.

**Methods and materials:**

We searched the PubMed, EMBASE and Cochrane Library databases until November 10, 2019. The methodological quality of the included studies was assessed by the Quality Assessment of Diagnostic Accuracy Studies 2 (QUADAS-2) tool. Bivariate modeling and hierarchical summary receiver operating characteristic (HSROC) modeling were performed to summarize and compare the diagnostic performance of blood/urine NGAL and serum cystatin C in CIN. Subgroup and meta-regression analyses were performed according to the study and patient characteristics.

**Results:**

Thirty-seven studies from thirty-one original studies were included (blood NGAL, 1840 patients in 9 studies; urine NGAL, 1701 patients in 10 studies; serum cystatin C, 5509 patients in 18 studies). Overall, serum cystatin C performed better than serum/urine NGAL (pooled DOR: 43 (95%CI: 12–152); AUROC: 0.93; λ: 3.79); serum and urine NGAL had a similar diagnostic performance (pooled DOR: 25 (95%CI: 6–108)/22(95%CI: 8–64); AUROC: 0.90/0.89; λ: 3.20/3.08). Meta-regression analysis indicated that the sources of heterogeneity might be CIN definition, assays, and nationalities.

**Conclusion:**

Both NGAL and cystatin C can serve as early diagnostic indicators of CIN, while cystatin C may perform better than NGAL.

## Introduction

Contrast-induced nephropathy (CIN) is defined as acute kidney injury (AKI) occurring 24–72 h after radiographic contrast media (CM) exposure in the absence of an alternative etiology[1]. After decreased renal perfusion (42%) and postoperative acute renal failure (18%), CIN is the third most common cause (12%) of hospital-acquired kidney failure[2, 3]. Half of the patients who develop CIN undergo cardiac catheterization and percutaneous coronary intervention (PCI)[2, 4]. CIN has become a major healthcare issue and is associated with adverse events, length of hospital stay and healthcare cost[5].

Currently, the diagnosis of CIN is based on the variation in serum creatinine (sCr) levels before and after CM exposure. However, sCr is a delayed and not always reliable indicator. After the kidneys undergo a contrast-induced toxicity attack, sCr typically increases within the first 24–48 h, peaks at 3–5 days and returns near baseline within 1–3 weeks[6]. The change in sCr is not evident until 50% of the nephrons have already been injured[7]. Furthermore, sCr can vary with many factors, such as age, sex, muscle mass, muscle metabolism, medications and hydration status[8]. Since there are so many limitations of sCr, the urgency for finding specific and sensitive biomarkers is highlighted. Besides, desirable biomarkers should also be rapidly quantifiable for analysis, which allows timely clinical interventions to be made[5].

Several promising biomarkers have been identified for the early diagnosis of CIN[1, 5]. Among them, neutrophil gelatinase-associated lipocalin (NGAL) and cystatin C are the most frequently investigated biomarkers in the clinic. NGAL is a 25-kDa protein covalently bound to gelatinase from secondary granules of human neutrophils and can reflect the damage of tubule cells[9, 10]. As the earliest biomarker after kidney injury, NGAL can be secreted and released into blood and urine in a short time and strongly correlates with sCr levels for CIN diagnosis[11]. After CM exposure, the levels of serum and urinary NGAL rise within 2 and 4 hours, respectively[12, 13]. Cystatin C is a 13-kDa cysteine proteinase inhibitor produced by nucleated cells and can be freely filtered by glomeruli, then reabsorbed and catabolized by the tubular cells[14]. It is less influenced by age, sex, race, muscle mass, steroid therapy, infection, liver disease or inflammation[15]. As cystatin C is merely distributed in the extracellular fluid volume and has a smaller distribution range than that of creatinine, serum cystatin C rises more rapidly than serum creatinine when GFR decreases[16–18]. Thus, serum cystatin C is a more accurate and earlier marker of GFR reduction than sCr.

Currently, multiple studies have reported that either NGAL or cystatin C alone could be viewed as a valuable predictor of early diagnosis for CIN; however, the comparison of the diagnostic performance between NGAL and cystatin C is still controversial and limited. Thus, we systemically reviewed relevant references and conducted a meta-analysis to summarize the predictive ability of serum/urine NGAL and serum cystatin C and to further compare those indicators on different occasions in order to provide significant evidence for the early diagnosis of CIN, which may provide more benefits for timely intervention and improvement of prognosis.

## Methods and materials

This systematic review and meta-analysis was performed according to the Cochrane Handbook for Systematic Reviews of Diagnostic Test Accuracy and reported according to the Preferred Reporting Items for Systematic Reviews and Meta-Analyses of Diagnostic Test Accuracy Studies (PRISMA-DTA) statement[19].

Two independent investigators (Yi He and Yunzhen Deng) conducted the “Data source” “Study selection” and “Data extraction and quality assessment” parts separately, and any disagreements were solved by discussion.

### Data source

PubMed, EMBASE and Cochrane Library databases were searched to identify possible references up to November 10, 2019. The electrical search strategy was developed based on the PICO format (P, patients/participants/population; I, index tests; C, comparator/reference tests; O, outcome), and search keywords were established using MeSH forms (PubMed) and Emtree forms (EMBASE).

The search terms are displayed as follows (Table 1).

**Table 1:** The search terms used in systematic review.

Frame	Search terms Diagnostic accuracy of NGAL versus cystatin C in contrast-induced nephropathy
**Population**	(contrast media) or (contrast agent) or (contrast materials) or (contrast material) or (radiocontrast media) or (radiocontrast agent) or (radiocontrast agents) or (radiopaque media)
**Index tests**	Lipocalin-2 or Lipocalin2 or (NGAL protein) or (oncogene 24p3 protein) or (siderocalin protein) or (neutrophil Gelatinase-Associated Lipocalin) or (neutrophil Gelatinase Associated Lipocalin) or (Lipocalin-2 protein) or (Lipocalin 2 protein)
**Comparator**	(Cystatin C) or (post-gamma-Globulin) or (post gamma Globulin) or (Neuroendocrine Basic Polypeptide) or (Cystatin 3) or (gamma-Trace) or (gamma Trace)
**Outcome**	(acute kidney injury) or (acute kidney injuries) or (acute renal injury) or (acute renal injuries) or (acute renal insufficiency) or (acute renal insufficiencies) or (acute kidney insufficiency) or (acute kidney failure) or (acute kidney failures) or (acute renal failure) or (acute renal failures) or (kidney disease) or (kidney diseases) or nephropathy

### Study selection

Raw data from separate databases were pooled in EndNote (version X9, Thomason Reuters Company) and screened to identify eligible studies. Duplicate records were removed. The inclusion and exclusion criteria were as follows.

### Inclusion criteria

1. Original clinical articles with adult participants (no restriction on prospective or retrospective studies).
2. Patients with suspected CIN or contrast-induced acute kidney injury.
3. NGAL (serum, plasma or urine source) or cystatin C performed as index tests.
4. Sufficient information to reconstruct a 2×2 table (sample capacity, sensitivity, and specificity, etc.).

### Exclusion criteria

1. Irrelevant article types: case reports, letters, replies, editorials, guidelines, consensus, conference abstracts, reviews, meta-analyses, or clinical trials.
2. Animal experiments.
3. Only reported the correlation between biomarkers and CIN/contrast-induced acute kidney injury.

### Data extraction and quality assessment

The characteristics and outcome data of the eligible studies were extracted according to the standardized form. The extracted data included study characteristics (first author, publication year, study region, study design, and CIN/contrast-induced AKI definition), patient characteristics (number of patients, age, sex distribution, baseline renal function, and settings) and index test characteristics (detection method of index, evaluation time, sample source, and cut-off value). A 2×2 table was constructed according to the study outcomes (true-positive (TP); true-negative (TN); false-positive (FP); and false-negative (FN) results). If only sensitivity and specificity were displayed in eligible studies, the 2×2 table would be created via the Bayesian method, with which the outcome data being back-calculated according to the sample capacity.

The methodological quality of the eligible studies was assessed by the Quality Assessment of Diagnostic Accuracy Studies 2 (QUADAS-2) tool[20]. The methodological quality graph and methodological quality summary were conducted by Review Manager (version 5.2. Copenhagen: The Nordic Cochrane Centre, The Cochrane Collaboration, 2012).

### Statistical analysis

The statistical analyses were performed using the “midas” and “metandi” modules in Stata software (version 14.2; StataCorp LP, College Station, TX) and Review Manager 5.2.

A mixed bivariate random-effects model was used for analyzing and pooling the diagnostic accuracy measurements across studies. We plotted the summary estimates of each test in forest plots and hierarchical summary receiver operating characteristic (HSROC) curves. The summary results are displayed as the 95% confidence region and 95% prediction region in the HSROC curve plot.

Heterogeneity was detected using the Cochrane Q test (P<0.05 indicates the presence of heterogeneity) and Higgins’ I^2^ test (heterogeneity can be roughly evaluated according to the value of I^2^ as follows: I^2^: 0–25%, might not be important; 25–50%, low heterogeneity; 50–75%, moderate heterogeneity; 75%-90%, high heterogeneity)[21]. The source of heterogeneity from the threshold effect can be assessed in three ways. The first way is to check the coupled forest plot of sensitivity and specificity (an inverse change in the side-by-side display of sensitivity and specificity in the forest plot indicates the presence of a threshold effect). The second way is to calculate the Spearman correlation coefficient between the sensitivity and false-positive rate (a coefficient >0.6 indicates a considerable threshold effect). The third way is to draw an SROC plot. The points in the plot showed an overall curvilinear distribution (from the lower-left corner to the upper right corner) in the ROC space, indicating the presence of a threshold effect[22].

Sensitivity analysis was conducted to examine the stability by omitting each study at a time to eliminate factors that influence heterogeneity. Meta-regression analyses using several covariates were conducted to explore the source of heterogeneity without the threshold effect.

Deeks funnel plot was performed to evaluate the publication bias (P value<0.1 indicates the presence of publication bias).

## Results

### Literature search and selection

**Fig 1:**
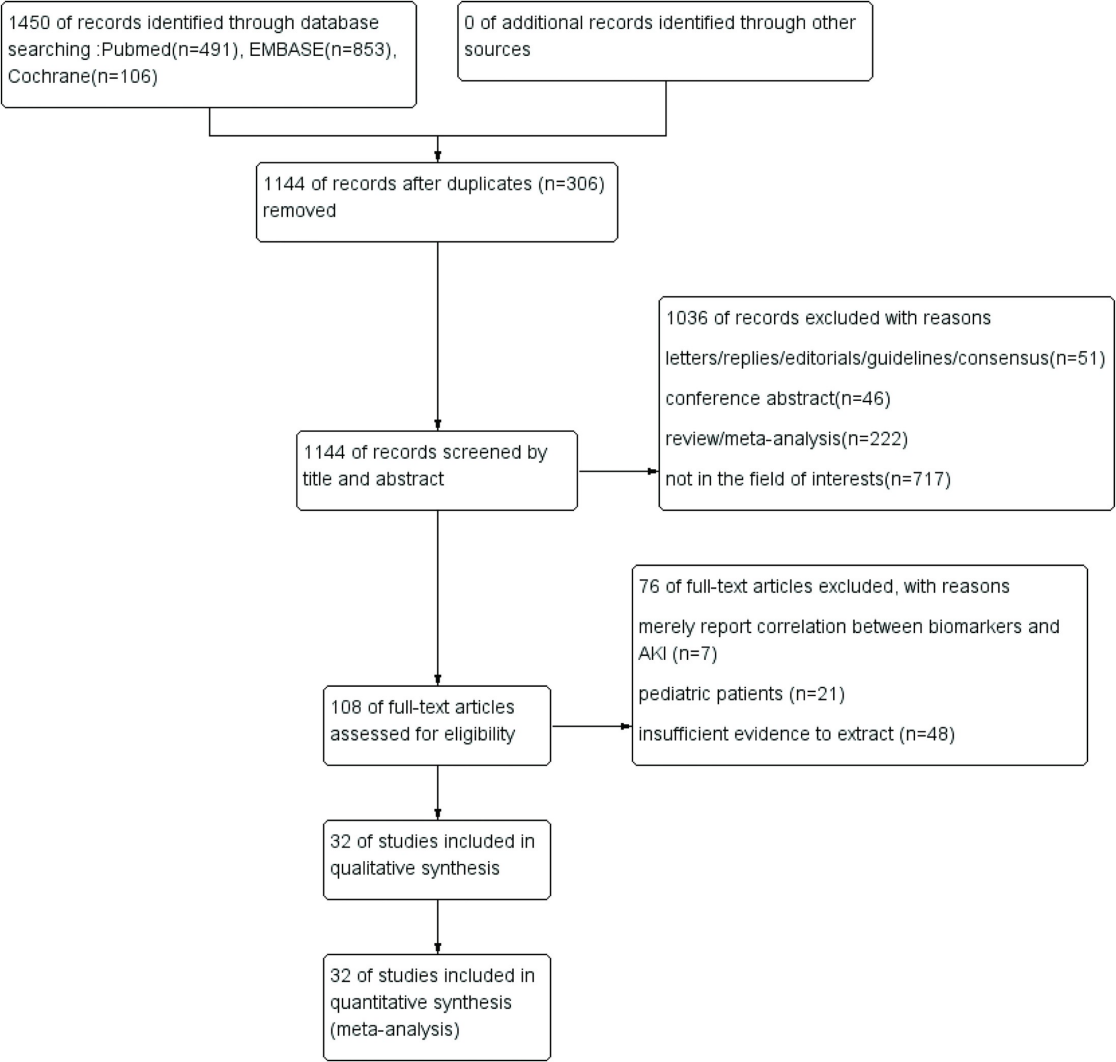
The process of study search and selection.

A diagram of the literature search and selection process is presented in Fig 1. The initial search from three databases identified 1450 relevant records. After removing 306 duplicate records, 1144 references were screened by title and abstract. A total of 1036 records were excluded for irrelevant article types and irrelevant content, e.g., randomized controlled trials and animal experiments. For the remaining 108 studies, the full texts were further assessed according to the inclusion criteria, and articles that contained pediatric patients, insufficient evidence or only reported correlations were removed. Finally, a total of 32 studies including 9088 patients were included in the quality assessment.

### Characteristics of the included studies

The included study characteristics, demographic features, and index test characteristics are summarized in Table 2 and Table 3.

**Table 2:** The characteristics of included study and population.

First Author	Year	Location	Study design	CIN definition	No. of patient	No. of CIN	Mean age^a^	Male(%)	Baseline sCr (mg/dL)^b^	Settings
Tasanarong A[23]	2013	Thailand	prospective	an increase of sCr above 0.3mg/dL or 1.5 times within 48 h	130	16	CIN:70±10; non-CIN:72±7	100(77)	CIN:2.00±0.60; non-CIN:1.40±0.40	undergoing CAG/PCI with eGFR ≤60 ml/min per 1.73m^2^ (except CKD 5)
Shukla AN[24]	2017	India	prospective	an increase of sCr above 0.5mg/dL or over 25% within 48h	253	31	56.54±10.04	206(81)	CIN:2.26±1.43; non-CIN:NR	undergoing CAG/PCI
Lacquaniti A[25]	2013	Italy	prospective	an increase of sCr above 0.5mg/dL or over 25%	60	23	men:57.7±11.3; women:60.6±12	30(50)	1.40±0.49	undergoing CM enhanced CT/MRI with CKD (30≤GFR ≤60 ml/min)
Liao B[26]	2019	China	prospective	an increase of sCr above 0.5mg/dL or over 25% within 72 h	240	25	60.92±6.38	128(53)	CIN:0.77±0.13; non-CIN:0.74±0.09	undergoing PCI
Briguori C[27]	2010	Italy	prospective	an increase of sCr above 0.3mg/dL at 48h	410	34	70±9	344(84)	1.64(1.51–1.90)	CAG/PAG/angioplasty procedure with CKD(eGFR ≤60 ml/min per 1.73m^2^)
Budano C[28]	2019	Italy	prospective	an increase of sCr above 0.3 mg/dL at 48h or over 50% in 7 days	713	47	66±11	520(73)	1.09±0.40	undergoing CAG
Quintavalle C[29]	2015	Italy	prospective	an increase of sCr above 0.3mg/dL at 48 h	458	64	CIN:74±9; non-CIN:75±8	302(66)	CIN:2.09(1.15–5.32); non-CIN:1.93(0.91–4.78)	undergoing CAG/PAG/angioplasty procedure with eGFR ≤30 ml/min per 1.73m^2^ or Mehran risk score≥11
Souza DF[30]	2015	Brazil	prospective	an increase of sCr above 0.3mg/dL at 48 h	125	22	CIN:60±10.8; non-CIN:62.5±10.3	63(50)	CIN:0.73±0.10; non-CIN:0.81±0.10	undergoing CAG
Cecchi E[31]	2017	Italy	prospective	an increase of sCr above 0.5mg/dL or over 25% within 48h	43	7	67.3±9.6	31(72)	0.85±0.17	undergoing PCI
Ribichini F[32]	2012	Italy	prospective	an increase of sCr 0.3–0.5mg/dL or over 25% within 48h	166	30	CIN:75 (64.3–79.8); non CIN:72.5(63.0–81.3)	120(72)	CIN:1.0 (0.77–1.50); non-CIN:1.02 (0.90–1.38)	undergoing CA/angioplasty
Kim GS[33]	2015	Korea	retrospective	an increase of sCr above 0.5mg/dL or over 25% within 48h	240	28	66.8±11.3	194(81)	1.20±0.60	undergoing PTA with intermittent claudication or critical limb ischemia
Li H[34]	2018	China	prospective	an increase of sCr above 0.5mg/dL or over 25% within 72 h	202	30	59.95±10.56	165(82)	CIN:1.09 (0.99–1.27); non-CIN:1.08 (0.96–1.22)	undergoing PCI
Torregrosa I[35]	2012	Spain	prospective	an increase of sCr over 50%	89	12	CIN:73±9; non-CIN:61±13	67(75)	CIN:1.20±0.30; non-CIN:0.94±0.22	undergoing CAG in ICU
Kato K[36]	2008	Japan	prospective	an increase of sCr above 0.5mg/dL or over 25% within 48h	87	18	67±11	62(71)	CIN:1.05±0.28; non-CIN:1.02±0.18	undergoing cardiac catheterization with/without PCI in CCU or ICU
Ning L[37]	2018	China	prospective	an increase of sCr over 50%	168	20	66.7±3.6	116(69)	CIN:0.89±0.09; non-CIN:0.96±0.07	undergoing PCI
LIU XL[38]	2012	China	prospective	an increase of sCr above 0.5mg/dL or over 25% within 48 h	311	39	CIN:63.2±10.5; non-CIN:58.4±9.3	198(64)	CIN:1.12±0.28; non-CIN:1.07±0.22	undergoing CAG/PCI with mild or moderate CKD
Connolly M[39]	2018	UK	prospective	an increase of sCr above 0.3mg/dL or over 50% within 48 h	301	28	CIN:69.9±10.1; non-CIN:73.9±8.0	170(56)	CIN:2.41±1.89; non-CIN:1.42±0.44	undergoing CAG with CKD (GFR ≤60 mls/min)
Khatami MR[40]	2015	Iran	prospective	an increase of sCr above 0.3mg/dL at 48 h	121	7	60±10.8	71(59)	0.90±0.20	undergoing CAG
Padhy M[41]	2014	India	nested case control	an increase of sCr above 0.5mg/dL or over 25% within 48–72 h	60	30	CIN:57.63±7.36; non-CIN:54.17±9.35	44(73)	CIN:0.86±0.24; non-CIN:0.82±0.19	undergoing PCI
Wang M[42]	2016	China	prospective	an increase of sCr above 0.5mg/dL or over 25% within 72 h	300	29	63.47±9.92	179(60)	CIN:0.87±0.16; non-CIN:0.91±0.12	undergoing CAG
Peng L[43]	2015	China	prospective	an increase of sCr above 0.5mg/dL or over 25% within 48h	196	29	70.4±11.3	134(68)	CIN:0.96±0.30; non-CIN:1.05±0.39	undergoing PCI
Xu Q[44]	2017	China	prospective	an increase of sCr above 0.5mg/dL or over 25% within 48–72 h or a rise in cystatin C over 25% within 3 days	213	52	52.07±14.52	164(77)	CIN:0.86±0.41; non-CIN:0.81±0.23	undergoing angiography
Alharazy SM[45]	2014	Malaysia	prospective	an increase of sCr over 25% within 48 h	100	11	60.4±8.3	79(79)	CIN:1.43±0.98; non-CIN:1.44±0.62	undergoing CAG with CKD (stage 2–4)
Li S(a)[46]	2015	China	prospective	an increase of sCr above 0.5mg/dL or over 25% within 48h	424	52	CIN:63.5±10.8; non CIN:65.4±10.4	244(58)	CIN:0.84±0.07; non-CIN:0.83±0.10	undergoing 320-slice CCTA
Li S(b)[47]	2015	China	prospective	an increase of sCr above 0.5mg/dL or over 25% within 48h	580	57	CIN:67.2±9.4; non CIN:62.6± 10.9	328(57)	CIN:0.94±0.06; non-CIN:0.93±0.09	undergoing 320-slice CCTA
Nozue T[48]	2010	Japan	prospective	an increase of sCr above 0.5mg/dL or over 25% within 48–72 h	96	5	70±10	73(76)	1.00±0.30	undergoing PCI
Wang L[49]	2014	China	prospective	an increase of sCr above 0.5mg/dL or over 25% within 72 h	42	14	CIN:60.2±9.5; non-CIN:60.6±8.1	23(55)	CIN:0.93±0.21; non-CIN:1.04±0.21	undergoing CAG or PCI
Ling W[50]	2008	China	prospective	an increase of sCr above 0.5mg/dL or over 25% within 48–72 h	40	13	CIN:66.3±9.9; non-CIN:68.62±10.6	24(60)	CIN:0.72±0.29; non-CIN:0.88±0.26	undergoing CAG
Zhang WF[51]	2017	China	prospective	an increase of sCr above 0.3mg/dL or over 50% within 48h	1071	25	64.8±10.2	713(67)	0.79 (0.67–0.94)	undergoing CAG or PCI
Valette X[52]	2013	France	prospective	an increase of sCr above 0.3mg/dL or over 50% within 72 h or <0.5 ml/kg/h of UO criteria over 6h	90	30	60(47–67)	74(82)	CIN:0.85(0.61–1.26); non-CIN:0.65(0.47–0.81)	undergoing imaging with CM administration (angiography and CT) in ICU
You W[53]	2016	China	prospective	an increase of sCr above 0.5mg/dL or over 25% within 48–72 h	506	47	CIN:65.3±10.9; non-CIN:64.2±10.5	319(63)	CIN:0.83±0.33; non-CIN:0.84±0.26	undergoing CAG or PCI

^a^ mean age ± standard deviation or median(interquartile range)

^b^ mean sCr ±standard deviation or median(interquartile range). sCr, serum creatinine; CIN, contrast-induced nephropathy; CKD, chronic kidney disease; CAG, coronary angiography; PCI, percutaneous coronary intervention; CM, contrast media; CT, computed tomography; MRI, magnetic resonance imaging; PAG, peripheral angiography; PTA, percutaneous transluminal angioplasty; eGFR, estimated glomerular filtration rate; ICU, intensive care unit; CCU, cardiac care unit; CCTA, coronary computed tomography angiography; NR, not report.

**Table 3:** Diagnostic value of blood NGAL, urine NGAL and serum cystatin C to predict CIN in each study.

First Author	Assay	source	Time of measurement	Cutoff	TP	FP	FN	TN	Sensitivity%(95%CI)	Specificity%(95%CI)	AUROC
**Blood NGAL**											
LIU XL	ELISA	Plasma	4h	80 ng/ml	20	53	19	219	96(80–100)	77(71–82)	0.662
Connolly M	biochips	Plasma	6h	1337 ng/ml	21	11	7	262	73(61–84)	52(47–57)	0.710
Valette X	Triage NGAL test	Plasma	24h	113 ng/ml	19	29	11	39	73(39–94)	77(67–85)	0.610
Lacquaniti A	Triage NGAL test	Serum	8h	115 ng/ml	23	5	0	32	51(35–68)	81(75–85)	0.995
Liao B	ELISA	Serum	12h	93.93 ng/ml	24	50	1	165	75(55–89)	96(93–98)	0.890
Quintavalle C	ELISA	Serum	6h	179 ng/ml	47	189	17	205	63(44–80)	57(45–69)	0.620
Li H	immunoturbidimetry	Serum	24h	111.5 ng/ml	26	64	4	108	100(85–100)	86(71–95)	0.779
Padhy M	ELISA	Serum	4h	155.2 ng/ml	30	1	0	29	87(69–96)	63(55–70)	1.000
Alharazy SM	ELISA	Serum	24h	increase of 17.7 ng/ml	8	23	3	76	100(88–100)	97(83–100)	0.845
**Urine NGAL**											
Tasanarong A	ELISA	urine	6h	117 ng/ml	15	25	1	89	94(70–100)	78(69–85)	0.850
Lacquaniti A	ELISA	urine	8h	90 ng/ml	22	1	1	36	96(78–100)	97(86–100)	0.992
Quintavalle C	ARCHITECT platform	urine	6h	20 ng/ml	48	189	16	205	75(63–85)	52(47–57)	0.610
Souza DF	ARCHITECT platform	urine	2h	increase of 50%	13	20	9	83	59(36–79)	81(72–88)	0.815
Torregrosa I	ELISA	urine	12h	31.9ng/ml	12	7	0	70	100(74–100)	91(82–96)	0.983
Ning L	ELISA	urine	2h	94.4 ng/mg of creatinine	15	27	5	121	75(51–91)	82(75–88)	0.632
Khatami MR	ELISA	urine	12h	22.5 ng/ml	5	48	2	66	71(29–96)	58(48–67)	0.533
Wang L	ELISA	urine	4h	11.95 ug/L	13	8	1	20	93(66–100)	71(51–87)	0.897
Ling W	ELISA	urine	24h	9.85 ng/ml	10	8	3	19	77(46–95)	70(50–86)	0.734
You W	nephelometry	urine	24h	increase of 4.65 ug/L	44	90	3	369	94(82–99)	80(76–84)	0.899
**Serum Cystatin C**											
Shukla AN	nephelometry	serum	24h	increase of 10%	31	49	0	173	100(89–100)	78(72–83)	0.901
Briguori C	particle-enhanced nephelometric immunoassay	serum	24h	increase of 10%	34	53	0	323	100(90–100)	86(82–89)	NR
Budano C	immunonephelometry	serum	0h	1.4 mg/L	30	107	17	559	64(49–77)	84(81–87)	0.820
Quintavalle C	NR	serum	24h	increase of 10%	27	43	37	351	42(30–55)	89(86–92)	0.660
Cecchi E	nephelometry	serum	0h	1.18ng/ml	6	8	1	28	86(42–100)	78(61–90)	0.863
Ribichini F	immunonephelometry	serum	12h	increase of 0.18 ng/ml	14	69	16	67	47(28–66)	49(41–58)	0.490
Kim GS	particle-enhanced nephelometric immunoassay	serum	0	1.35mg/L	21	42	7	170	75(55–89)	80(74–85)	0.757
Torregrosa I	nephelometric immunoassay	serum	12h	0.8mg/L	11	18	1	59	92(62–100)	77(66–86)	0.869
Kato K	particle-enhanced nephelometric immunoassay	serum	NR	1.2mg/L	17	10	1	59	94(73–100)	86(75–93)	0.933
Padhy M	ELISA	serum	24h	0.994mg/L	30	1	0	29	100(88–100)	97(83–100)	1.000
Wang M	NR	serum	24h	1.55mg/L	24	6	5	265	83(64–94)	98(95–99)	0.928
Peng L	particle-enhanced colorimetric immunoassay	serum	48h	increase of 15%	12	12	17	155	41(24–61)	93(88–96)	0.783
Xu Q	particle-enhanced colorimetric immunoassay	serum	48h	1.605mg/L	48	76	4	85	92(81–98)	53(45–61)	0.715
Alharazy SM	particle-enhanced nephelometric immunoassay	serum	24h	increase of 0.19mg/L	7	11	4	88	64(31–89)	89(81–94)	0.800
Li S (a)	immunoturbidimetric	serum	48h	1.61mg/dL	52	0	0	372	100(93–100)	100(99–100)	1.000
Li S (b)	immunoturbidimetric	serum	0	1.05mg/dL	39	148	18	375	68(55–80)	72(68–76)	0.774
Nozue T	particle-enhanced nephelometric immunoassay	serum	0h	1.26mg/L	4	25	1	66	80(28–99)	73(62–81)	0.825
Zhang WF	particle-enhanced nephelometric immunoassay	serum	48h	increase of 15%	20	178	5	868	80(59–93)	83(81–85)	0.856

TP, true positive; FP, false positive; FN, false negative; TN, true negative; 95%CI, 95% confidence interval; AUROC, area under receiver operating characteristics curve; ELISA, enzyme linked immunosorbent assay; NR, not report.

Most of the included studies were prospective studies (n = 30), while one of the remaining studies was a retrospective study and the other was a nested case-control study. Among them, most studies were performed in patients undergoing percutaneous coronary intervention (PCI)/coronary angiography (CAG). The diagnostic performance of blood NGAL, urine NGAL and serum cystatin C for contrast-induced nephropathy (CIN) was reported in 9, 10 and 18 studies, respectively.

### Quality assessment

**Fig 2:**
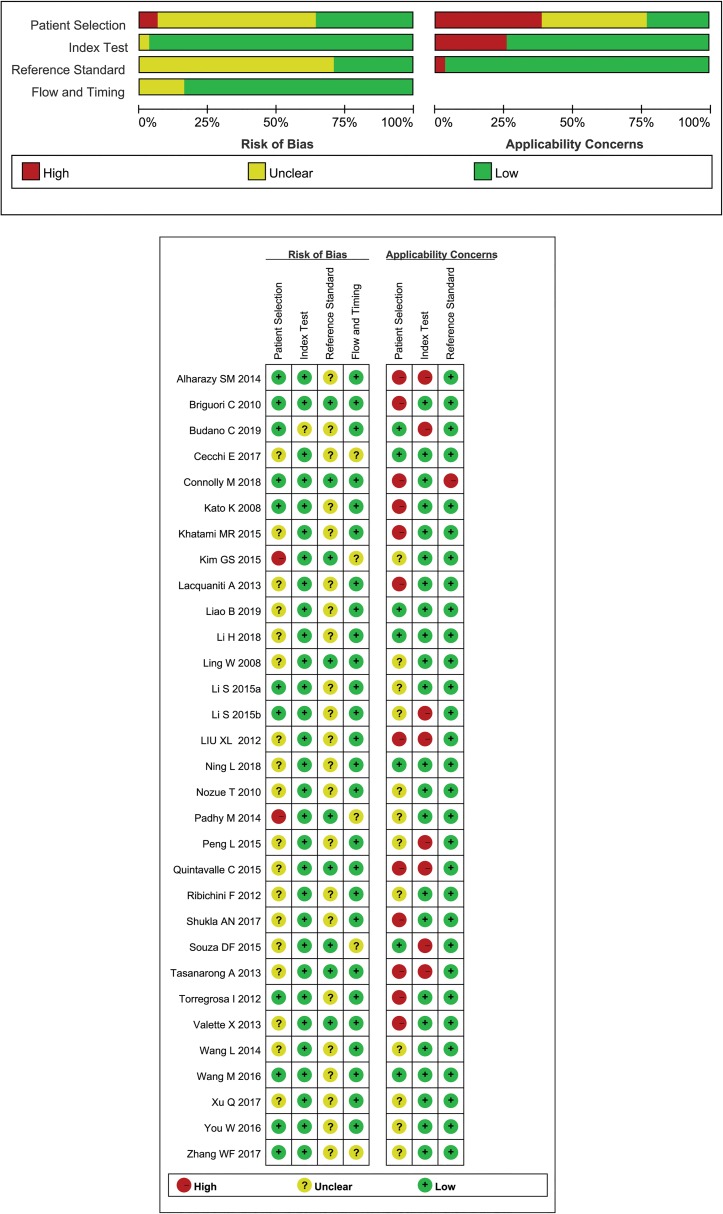
The methodological quality assessment. The methodological quality of included studies was assessed according to the Quality Assessment of Diagnostic Accuracy Studies 2 (QUADAS-2) tool.

The risk of bias and applicability concerns for the 32 included studies are shown in Fig 2. The methodological quality in all included studies was relatively high, which meant that each study satisfied at least 4 items.

Regarding the patient selection and reference standard domains, over 50% of studies were considered to have a relatively high risk of bias for heterogeneity because of the complex patients’ source and unfixed definition of CIN.

### Diagnostic performance

#### Blood NGAL

**Fig 3:**
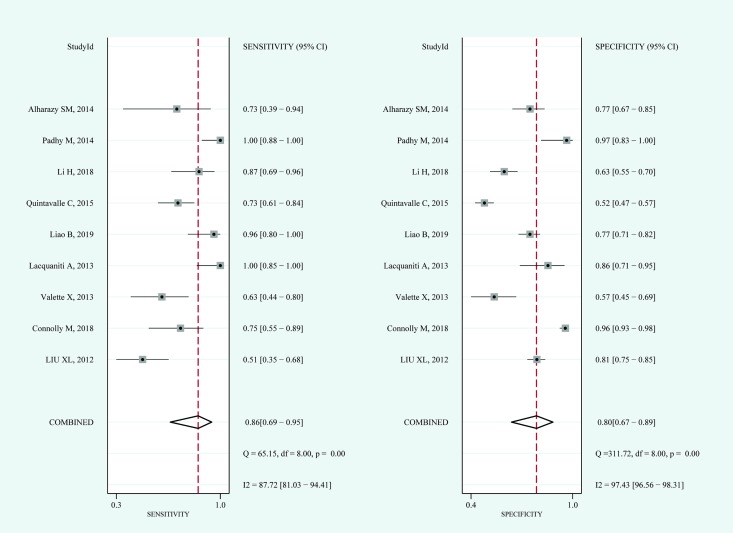
Coupled forest plots for the pooled sensitivity and specificity of blood NGAL for the diagnosis of CIN. Dots in squares represent sensitivity and specificity. Horizontal lines represent the 95% confidence interval (CI) for each included study. The pooled estimate is based on the random-effects model. Heterogeneities evaluation, I^2^ with 95% CIs and Q are provided. Q is Cochrane heterogeneity statistic and df is the degrees of freedom.

For blood NGAL, the pooled sensitivity and specificity were 0.86 (95%CI: 0.69–0.95) and 0.80 (95%CI: 0.67–0.89), respectively (Fig 3). The pooled diagnostic odds ratio was 25 (95%CI: 6–108). The area under the summary receiver operating characteristic curve (AUROC) of blood NGAL was 0.90. The Q test indicated significant heterogeneity (P = 0.000); I^2^ tests in sensitivity (I^2^ = 87.72%) and specificity (I^2^ = 97.43%) also demonstrated high heterogeneity. After a visual analysis of the distribution of the coupled forest plot and calculation of the correlation coefficient (0.44), the results showed that there was no significant threshold effect. The results of blood NGAL in the hierarchical summary receiver operating characteristic model were β = -0.27 (95%CI:-1.14–0.59, Z = -0.62, P = 0.538), which reflected that the shape of the SROC curve was symmetric; and λ = 3.20, which indicated that the diagnostic accuracy of blood NGAL for CIN was moderate.

#### Urine NGAL

For urine NGAL, the pooled diagnostic odds ratio (DOR) was 22 (95%CI: 8–64). The AUROC of urine NGAL was 0.89. The Q test did not indicate significant heterogeneity (P = 0.06), while I^2^ tests still demonstrated moderate heterogeneity (I^2^ = 52%). There was a significant threshold effect since the correlation coefficient was 0.69. The results of urine NGAL in the hierarchical summary receiver operating characteristic model were β = -0.14 (95%CI: -1.01–0.73, Z = -0.31, P = 0.753), which reflected that the shape of the SROC curve was symmetric; and λ = 3.08, which indicated that the diagnostic value of urine NGAL for CIN was moderate.

#### Serum cystatin C

**Fig 4:**
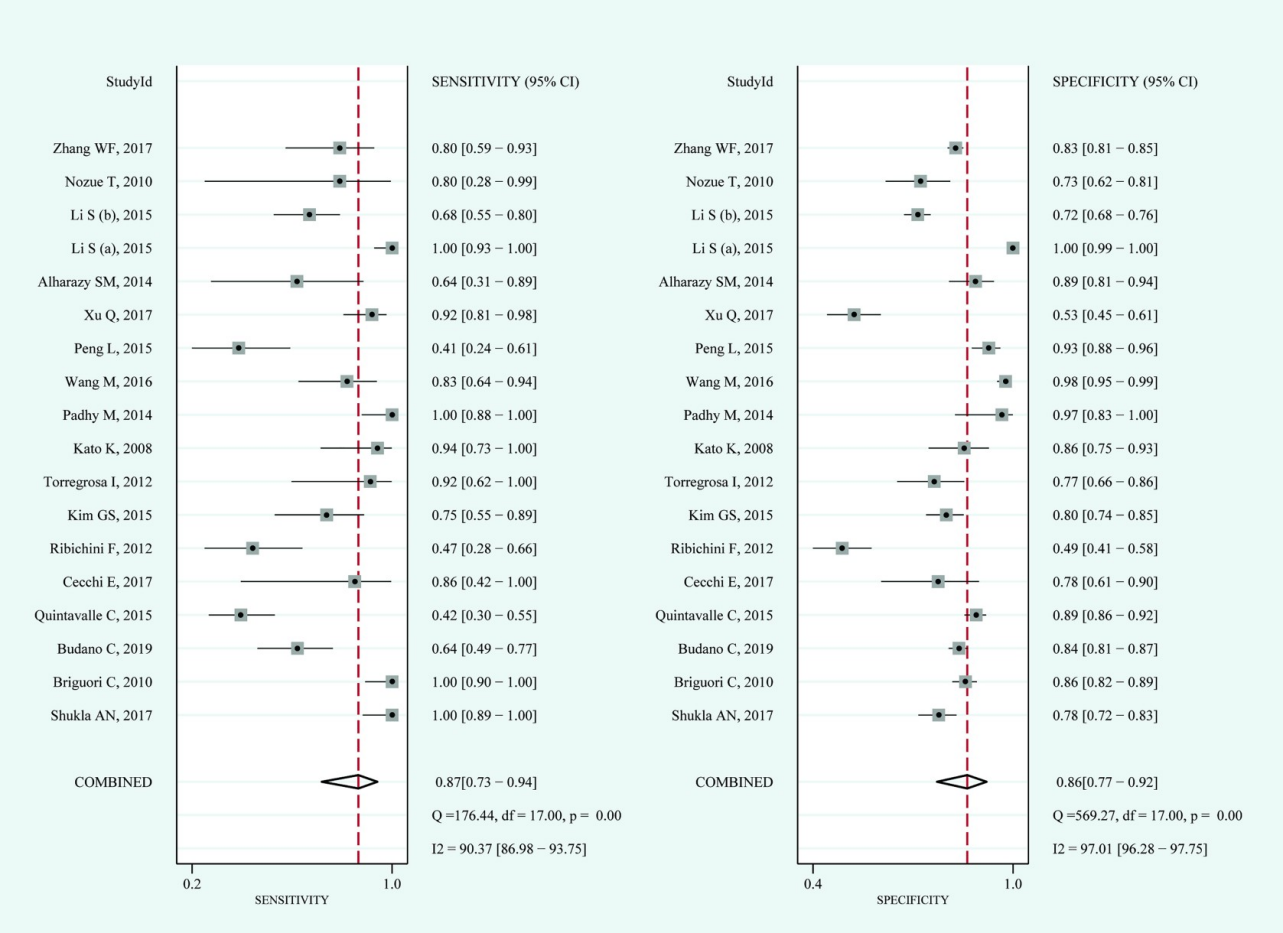
Coupled forest plots for the pooled sensitivity and specificity of serum cystatin C for the diagnosis of CIN. Dots in squares represent sensitivity and specificity. Horizontal lines represent the 95% confidence interval (CI) for each included study. The pooled estimate is based on the random-effects model. Heterogeneities evaluation, I^2^ with 95% CIs and Q are provided. Q is Cochrane heterogeneity statistic and df is the degrees of freedom.

For serum cystatin C, the pooled sensitivity and specificity were 0.87 (95%CI: 0.73–0.94) and 0.86 (95%CI: 0.77–0.92), respectively (Fig 4). The pooled diagnostic odds ratio was 43 (95%CI: 12–152). The AUROC of serum cystatin C was 0.93. The Q test indicated significant heterogeneity (P = 0.000); I^2^ tests in sensitivity (I^2^ = 90.37%) and specificity (I^2^ = 97.01%) also demonstrated high heterogeneity. After a visual analysis of the distribution of the coupled forest plot and calculation of the correlation coefficient (0.41), the results showed that there was no significant threshold effect. The results of serum cystatin C in the hierarchical summary receiver operating characteristic model were β = -0.28 (95%CI:-0.88–0.31, Z = -0.93, P = 0.352); and λ = 3.79, which indicated that the diagnostic value of blood NGAL for CIN was moderate.

#### Comparison of blood NGAL, urine NGAL and serum cystatin C

Test comparisons of the diagnostic performance for CIN among blood NGAL, urine NGAL and serum cystatin C were conducted.

**Fig 5:**
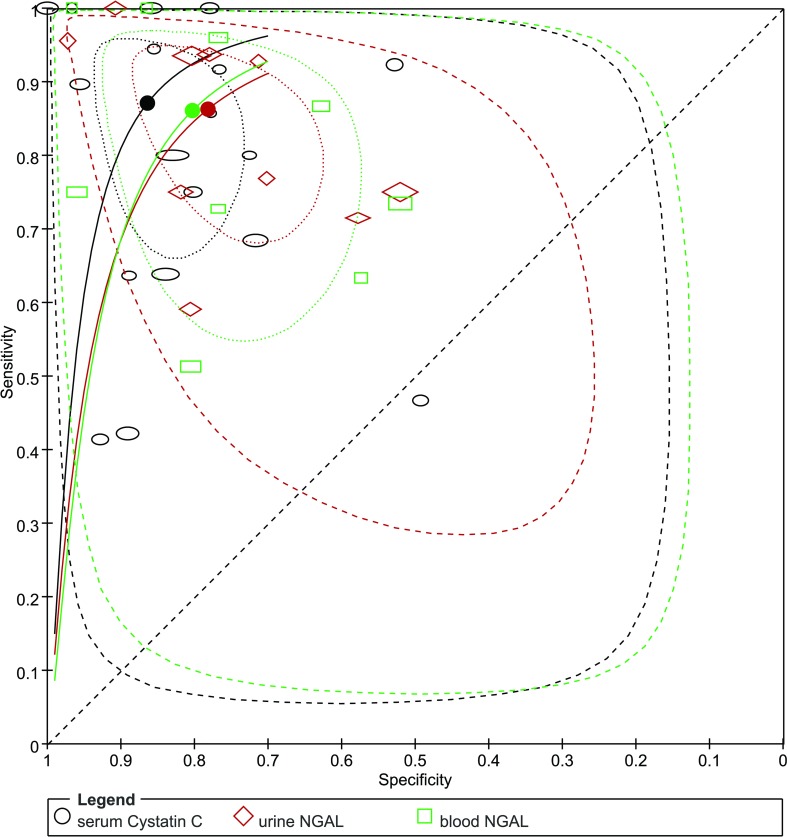
Hierarchical summary receiver operating characteristic (HSROC) curve for blood NGAL, urine NGAL and serum cystatin C for the diagnosis of CIN. The black, green and red dots present the summary points for serum cystatin C, blood NGAL and urine NGAL respectively. The area circled by dot-dashed lines represent 95% confidence region; the area circled by dashed lines represent 95% prediction region.

Overall, the results of the summary AUROC, DOR and λ suggested that serum cystatin C may perform better than blood NGAL and urine NGAL in diagnosing CIN. The comparison of HSROC curves is shown in Fig 5.

We compared the diagnostic accuracy of blood NGAL, urine NGAL and serum cystatin C at different cut-off times. The subgroup analysis results are shown in Table 4. The results indicated that blood NGAL may perform better than urine NGAL within 6 h after contrast media exposure; however, after 6 h, urine NGAL might be a better predictor of CIN than blood NGAL. For serum cystatin C, when measuring the level of cystatin C within 24 h after the procedure, the predictive performance was better than that at baseline.

**Table 4:** Subgroup analysis of diagnostic performance for index tests in different measuring time.

Subgroups	No. of studies	Sensitivity%(95%CI)^a^	Specificity%(95%CI)^a^	DOR	AUROC	95%CI
**blood NGAL**						
<6h	4	-	-	35	0.92	0.89–0.94
>6h	5	-	-	23	0.84	0.81–0.87
**urine NGAL**		-	-			
<6h	5	78(64–88)	74(62–82)	10	0.83	0.79–0.86
>6h	5			53	0.94	0.91–0.95
**serum cystatin C**						
0h(baseline)	5	-	-	8	0.75	0.71–0.79
<24h	8	93(65–99)	86(75–92)	77	0.93	0.90–0.95

NGAL, neutrophil gelatinase-associated lipocalin; DOR, diagnostic odds ratio; AUROC, area under the summary receiver operating characteristic curve; 95% CI, 95% confidence interval. ^a^ Owing to the threshold effect, pooled sensitivity and specificity for some subgroups could not be calculated and presented as the absence of value.

### Sensitivity analysis and meta-regression analyses

Using Cook’s distance, the sensitivity analysis showed particularly influential observations in the blood NGAL (studies from Connolly M, Padhy M), urine NGAL (study from Souza DF) and serum cystatin C (study from Li S(a)) groups.

The meta-regression analysis results are shown in the S1 Table. Among them, the significant sources of heterogeneity were “CIN definition time”, “assay” and “sample source” for the blood NGAL group; and “CIN definition time” and “location” for the urine NGAL group. In the serum cystatin C group, there was no significant source of heterogeneity initially. However, after omitting the most particular influential study, the significant source of heterogeneity came from the “assay” of detecting serum cystatin C. Other covariates were not significantly responsible for the heterogeneity between the studies.

### Publication bias

**Fig 6:**
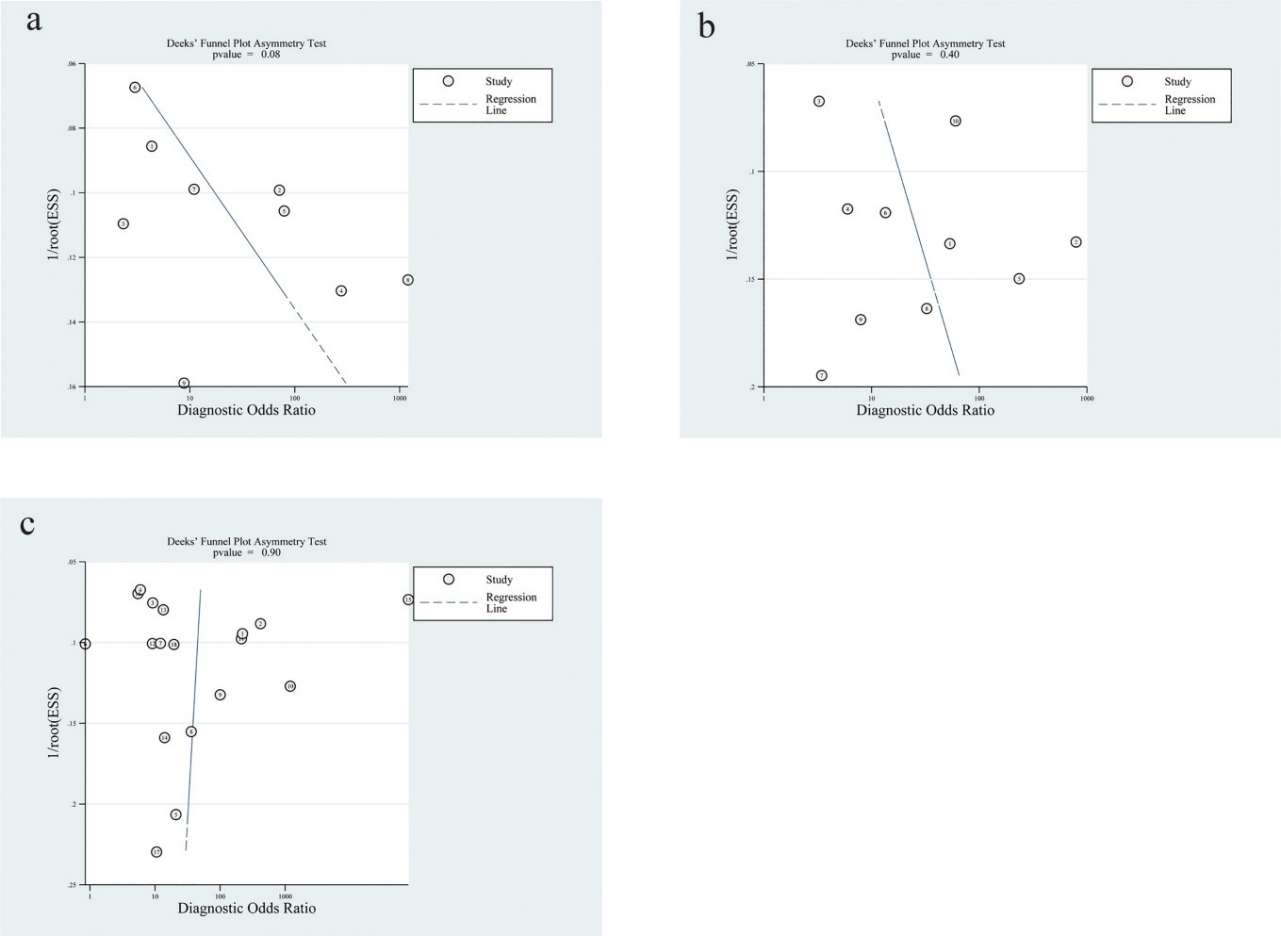
Deek’s funnel plot asymmetry test for publication bias of blood NGAL(a), urine NGAL(b) and serum cystatin C(c). There was no considerable publication heterogeneity in each group.

Deek’s test showed that there was no significant publication bias in each group (P value = 0.08 for blood NGAL, 0.40 for urine NGAL and 0.90 for serum cystatin C) (Fig 6).

## Discussion

Owing to the constant shortcomings of serum creatinine for the early diagnosis of CIN, NGAL and cystatin C have been regarded as promising biomarkers in clinical practice. Our results suggested the following: 1) overall, the diagnostic performance of serum cystatin C is better than that of blood NGAL and urine NGAL; 2) blood and urine NGAL have similar predictive value, while the diagnostic accuracies of blood NGAL and urine NGAL were opposite within or beyond 6 h after CM exposure; and 3) serum cystatin C after CM exposure performed better in predicting CIN compared with that at baseline.

The increase in cystatin C and NGAL levels could represent a reduction in the glomerular filtration rate and renal damage, respectively. As a low molecular weight protein, cystatin C could be freely filtered by glomeruli and completely reabsorbed and catabolized by renal tubules on normal occasions. After kidney injury, the rise of cystatin C is much earlier and superior to sCr in detecting reduced glomerular filtration rate (GFR)[54, 55]. In CIN patients, serum cystatin C was shown to peak mainly at 24 h after CM administration, which is delayed compared with the rise in serum/urine NGAL levels[56]. NGAL can be viewed as the most rapid indicator after renal tubular injuries. After iodine toxicity injury occurs in tubular cells, they secrete more NGAL than normal in response to nephrotoxic or ischemic stimuli, and the reabsorption ability of the proximal tubule is decreased. Both mechanisms contribute to the rise in serum/urine NGAL levels[57, 58]. However, NGAL can be secreted by other tissues and activated by neutrophils as an acute-phase protein, which constitutes important confounders. Cecchi E et al. [31] demonstrated that serum cystatin C was associated with serum creatinine and the occurrence of CIN in patients undergoing percutaneous coronary invasive procedures (PCIPs). The rise in NGAL may suggest injury not only from the kidney but also from acute/chronic inflammation, especially in patients in intensive care settings. Singer E et al. [58] also indicated that NGAL would not be accurate enough in predicting AKI in patients with nonrenal diseases. In our results, owing to the threshold effect, we could not directly compare the pooled sensitivity and specificity between urine NGAL and serum cystatin C, while the operating points of serum NGAL and serum cystatin C were similar. Nevertheless, the summary ROC indicated that the diagnostic performance of serum cystatin C was the most valuable compared with the other two indicators. It could be interpreted that serum cystatin C, regardless of the baseline level or increases after CM exposure, could be a good predictive indicator for CIN, while NGAL is more likely to be influenced by other factors.

Several studies indicated that the rise in urine NGAL occurs a few hours later than that of blood NGAL[56, 59, 60]. Bachorzewska-Gajewska H et al.[56] demonstrated that serum and urine NGAL significantly increased at 2 and 4 hours after CM exposure, respectively, and Malyszko J et al.[60] also found that the peak of serum and urine NGAL was at 4 and 8 hours in patients undergoing cardiac catheterization. However, studies from Lacquaniti A et al.[25] and Quintavalle C et al.[29] reported that serum and urine NGAL have similar value in predicting the incidence of CIN, and our summary estimates also confirmed this view. We further investigated the diagnostic performance of serum/urine NGAL in different phases. The results indicated that blood NGAL performed well in the early phase (within 6 hours after the procedure), while the diagnostic performance of urinary NGAL was better than that of blood NGAL beyond 6 hours, which conformed to the time-course change of NGAL in serum/urine.

People with high-risk factors, such as chronic kidney disease, diabetes mellitus, dehydration, poor cardiac function, advanced age, anemia, and contrast media volume, are more likely to develop CIN[61, 62]. Among them, pre-existing CKD is the most important risk factor for CIN, and the level of serum cystatin C is higher in patients with insufficient kidney function than in the normal population[63–65]. Thus, a high level of baseline cystatin C could be seen as a predictor of high-risk populations for CIN. However, regarding the diagnostic performance of CIN, the increase in cystatin C after CM administration is better and more accurate than that at baseline.

According to those results and analyses, we proposed that it seems reasonable to combine serum cystatin C, blood NGAL and urine NGAL for diagnosing CIN. Cystatin C and NGAL have their benefits and limitations as early predictors. In clinical practice, desirable biomarkers should be sensitive and convenient to monitor in order to supply timely support; however, it should also avoid the overdiagnosis pitfall. Furthermore, it is difficult for a single marker to supply functional and damage information at the same time[5, 66]. The instant renal injury and decreased renal filtration rate can be reflected by the variation in NGAL and cystatin C, respectively, while the possibility that nonrenal factors affect CIN diagnosis would be reduced. However, care must be taken in combining biomarkers, and further investigation is needed before application in clinical practice.

There was moderate or high heterogeneity in each group since the designs and result interpretation were not standard across studies. To explore the source of heterogeneity, we further conducted subgroup and meta-regression analyses for blood/urine NGAL and serum cystatin C. First, there was no significant difference between CKD patients and other populations. We chose CKD patients as a high-risk population because other risk factors were complex and confounded. NGAL and cystatin C could be applied in different populations. Second, it is evident that diverse CIN definitions hamper the comparison across studies. According to the diagnostic criteria from European Society of Urogenital Radiology (ESUR)[67] and Acute Kidney Injury Network (AKIN) [68], the endpoints of CIN are absolute increase of sCr of 0.5mg/dL and 0.3mg/dL or relative increase of sCr of 25% and 50% respectively. Meanwhile, the time limits are also different, within 72h and 48h separately. Based on our results, the cut-off value of the CIN definition was not responsible for the heterogeneity, but timepoint significantly influenced the diagnostic performance of NGAL. Third, when summarizing estimates, the comparability of assays for individual biomarkers should be taken into account. Assays applied in blood NGAL and cystatin C were also significant sources of heterogeneity. For NGAL, the concentrations were significantly different when using different methods[69], and the concentration of NGAL was not equivalent in plasma and serum[70]. There were also discrepancies in the diagnostic performance of cystatin C in different assays relating to the source of antibodies or different instruments[71–74]. Fourth, care should be taken in explaining the result that the diagnostic accuracy of urine NGAL was influenced by race/nationality. Only a study from Brazil[30] was responsible for the heterogeneity in the urine NGAL group. However, there was no significant difference in the diagnostic accuracy of NGAL/cystatin C between European and Asian nationalities (the results are not listed in the S1 Table).

The strength of our study is that we extensively collected studies from different countries and locations and utilized available information regarding the performance of NGAL and cystatin C in predicting CIN. Unfortunately, there are still some limitations to our study. First, we did not provide a cut-off value for separate index tests. Second, the diagnostic accuracy for the combination of cystatin C and NGAL needs further investigation. Third, the designs of the included studies were totally different and complex. Even if we enforced strict inclusion criteria and set covariates for meta-regression analysis in advance, there were still sources of heterogeneity we cannot completely explain.

In conclusion, both NGAL and cystatin C can serve as early diagnostic indicators of CIN. The combination of NGAL and cystatin C is likely to provide more diagnostic information, but more evidence is still needed.

## Supporting information

S1 Checklist PRISMA 2009 checklist. (DOCX)

S1 Table Meta-regression analyses for potential sources of heterogeneity from each group. (DOCX)

## References

[1] Andreucci M, Faga T, Riccio E, Sabbatini M, Pisani A, Michael A. The potential use of biomarkers in predicting contrast-induced acute kidney injury. Int J Nephrol Renovasc Dis. 2016;9:205–221. 10.2147/IJNRD.S105124 27672338 PMC5024777

[2] Nash K, Hafeez A, Hou S. Hospital-acquired renal insufficiency. Am J Kidney Dis. 2002;39:930–936. 10.1053/ajkd.2002.32766 11979336

[3] Hou SH, Bushinsky DA, Wish JB, Cohen JJ, Harrington JT. Hospital-acquired renal insufficiency: a prospective study. Am J Med. 1983;74:243–248. 10.1016/0002-9343(83)90618-6 6824004

[4] Lloyd-Jones D, Adams RJ, Brown TM, Carnethon M, Dai S, De Simone G, et al Heart disease and stroke statistics—2010 update: a report from the American Heart Association. Circulation. 2010;121:e46–e215. 10.1161/CIRCULATIONAHA.109.192667 20019324

[5] Connolly M, McEneaney D, Menown I, Morgan N, Harbinson M. Novel Biomarkers of Acute Kidney Injury After Contrast Coronary Angiography. Cardiol Rev. 2015;23:240–246. 10.1097/CRD.0000000000000058 25699983

[6] Mehran R, Nikolsky E. Contrast-induced nephropathy: definition, epidemiology, and patients at risk. Kidney Int Suppl. 2006;69:S11–15. 10.1038/sj.ki.5000368 16612394

[7] Mussap M, Noto A, Fanos V, Van Den Anker JN. Emerging biomarkers and metabolomics for assessing toxic nephropathy and acute kidney injury (AKI) in neonatology. Biomed Res Int. 2014;2014:602526 10.1155/2014/602526 25013791 PMC4071811

[8] Devarajan P. Neutrophil gelatinase-associated lipocalin (NGAL): a new marker of kidney disease. Scand J Clin Lab Invest Suppl. 2008;241:89–94. 10.1080/00365510802150158 18569973 PMC2528839

[9] Kjeldsen L, Johnsen AH, Sengelov H, Borregaard N. Isolation and primary structure of NGAL, a novel protein associated with human neutrophil gelatinase. J Biol Chem. 1993;268:10425–10432. 7683678

[10] Filiopoulos V, Biblaki D, Vlassopoulos D. Neutrophil gelatinase-associated lipocalin (NGAL): a promising biomarker of contrast-induced nephropathy after computed tomography. Ren Fail. 2014;36:979–986. 10.3109/0886022X.2014.900429 24673459

[11] Haase M, Bellomo R, Devarajan P, Schlattmann P, Haase-Fielitz A. Accuracy of neutrophil gelatinase-associated lipocalin (NGAL) in diagnosis and prognosis in acute kidney injury: a systematic review and meta-analysis. Am J Kidney Dis. 2009;54:1012–1024. 10.1053/j.ajkd.2009.07.020 19850388

[12] Devarajan P. Neutrophil gelatinase-associated lipocalin: a promising biomarker for human acute kidney injury. Biomark Med. 2010;4:265–280. 10.2217/bmm.10.12 20406069 PMC2893148

[13] Bachorzewska-Gajewska H, Malyszko J, Sitniewska E, Malyszko JS, Dobrzycki S. Neutrophil-gelatinase-associated lipocalin and renal function after percutaneous coronary interventions. Am J Nephrol. 2006;26:287–292. 10.1159/000093961 16772710

[14] Stacul F, Adam A, Becker CR, Davidson C, Lameire N, McCullough PA, et al Strategies to reduce the risk of contrast-induced nephropathy. Am J Cardiol. 2006;98:59k–77k. 10.1016/j.amjcard.2006.01.024 16949381

[15] Zhu J, Yin R, Wu H, Yi J, Luo L, Dong G, et al Cystatin C as a reliable marker of renal function following heart valve replacement surgery with cardiopulmonary bypass. Clin Chim Acta. 2006;374:116–121. 10.1016/j.cca.2006.06.001 16876777

[16] Tenstad O, Roald AB, Grubb A, Aukland K. Renal handling of radiolabelled human cystatin C in the rat. Scand J Clin Lab Invest. 1996;56:409–414. 10.3109/00365519609088795 8869663

[17] Schloerb PR. Total body water distribution of creatinine and urea in nephrectomized dogs. Am J Physiol. 1960;199:661–665. 10.1152/ajplegacy.1960.199.4.661 13747834

[18] Briguori C, Quintavalle C, Donnarumma E, Condorelli G. Novel biomarkers for contrast-induced acute kidney injury. Biomed Res Int. 2014;2014:568738 10.1155/2014/568738 24982897 PMC4058136

[19] Frank RA, Bossuyt PM, McInnes MDF. Systematic Reviews and Meta-Analyses of Diagnostic Test Accuracy: The PRISMA-DTA Statement. Radiology. 2018;289:313–314. 10.1148/radiol.2018180850 30015590

[20] Whiting PF, Rutjes AW, Westwood ME, Mallett S, Deeks JJ, Reitsma JB, et al QUADAS-2: a revised tool for the quality assessment of diagnostic accuracy studies. Ann Intern Med. 2011;155:529–536. 10.7326/0003-4819-155-8-201110180-00009 22007046

[21] Higgins JP, Thompson SG, Deeks JJ, Altman DG. Measuring inconsistency in meta-analyses. BMJ. 2003;327:557–560. 10.1136/bmj.327.7414.557 12958120 PMC192859

[22] Kim KW, Lee J, Choi SH, Huh J, Park SH. Systematic Review and Meta-Analysis of Studies Evaluating Diagnostic Test Accuracy: A Practical Review for Clinical Researchers-Part I. General Guidance and Tips. Korean J Radiol. 2015;16:1175–1187. 10.3348/kjr.2015.16.6.1175 26576106 PMC4644738

[23] Tasanarong A, Hutayanon P, Piyayotai D. Urinary Neutrophil Gelatinase-Associated Lipocalin predicts the severity of contrast-induced acute kidney injury in chronic kidney disease patients undergoing elective coronary procedures. BMC Nephrol. 2013;14:270 10.1186/1471-2369-14-270 24305547 PMC4234212

[24] Shukla AN, Juneja M, Patel H, Shah KH, Konat A, Thakkar BM, et al Diagnostic accuracy of serum cystatin C for early recognition of contrast induced nephropathy in Western Indians undergoing cardiac catheterization. Indian Heart J. 2017;69:311–315. 10.1016/j.ihj.2016.12.010 28648419 PMC5485381

[25] Lacquaniti A, Buemi F, Lupica R, Giardina C, Murè G, Arena A, et al Can neutrophil gelatinase-associated lipocalin help depict early contrast material-induced nephropathy? Radiology. 2013;267:86–93. 10.1148/radiol.12120578 23297321

[26] Liao B, Nian W, Xi A, Zheng M. Evaluation of a Diagnostic Test of Serum Neutrophil Gelatinase-Associated Lipocalin (NGAL) and Urine KIM-1 in Contrast-Induced Nephropathy (CIN). Med Sci Monit. 2019;25:565–570. 10.12659/MSM.912569 30659575 PMC6347915

[27] Briguori C, Visconti G, Rivera NV, Focaccio A, Golia B, Giannone R, et al Cystatin C and contrast-induced acute kidney injury. Circulation. 2010;121:2117–2122. 10.1161/CIRCULATIONAHA.109.919639 20439784

[28] Budano C, Andreis A, Filippo OD, Bissolino A, Lanfranco G, Usmiani T, et al A single cystatin C determination before coronary angiography can predict short and long-term adverse events. Int J Cardiol. 2019; 300:73–79. 10.1016/j.ijcard.2019.09.069 31619362

[29] Quintavalle C, Anselmi CV, De Micco F, Roscigno G, Visconti G, Golia B, et al Neutrophil Gelatinase-Associated Lipocalin and Contrast-Induced Acute Kidney Injury. Circ Cardiovasc Interv. 2015;8:e002673 10.1161/CIRCINTERVENTIONS.115.002673 26333343

[30] Souza DF, Reis SS, Botelho RV, Ferreira-Filho SR. Relative and absolute changes in urinary neutrophil gelatinase-associated lipocalin and correlation with small increases in serum creatinine levels after coronary angiography: an observational study. Nephron. 2015;129:84–90. 10.1159/000368413 25662930

[31] Cecchi E, Avveduto G, D'Alfonso MG, Terreni A, Gelera E, Caldini A, et al Cystatin C, but not urinary or serum NGAL, may be associated with contrast induced nephropathy after percutaneous coronary invasive procedures: A single center experience on a limited number of patients. Acta Med Acad. 2017;46:34–43. 10.5644/ama2006-124.184 28605926

[32] Ribichini F, Gambaro G, Graziani MS, Pighi M, Pesarini G, Pasoli P, et al Comparison of serum creatinine and cystatin C for early diagnosis of contrast-induced nephropathy after coronary angiography and interventions. Clin Chem. 2012;58:458–464. 10.1373/clinchem.2011.170464 22166252

[33] Kim GS, Ko YG, Shin DH, Kim JS, Kim BK, Choi D, et al Elevated serum cystatin C level is an independent predictor of contrast-induced nephropathy and adverse outcomes in patients with peripheral artery disease undergoing endovascular therapy. J Vasc Surg. 2015;61:1223–1230. 10.1016/j.jvs.2014.11.079 25595408

[34] Li H, Yu Z, Gan L, Peng L, Zhou Q. Serum NGAL and FGF23 may have certain value in early diagnosis of CIN. Ren Fail. 2018;40:547–553. 10.1080/0886022X.2018.1487860 30278796 PMC6171456

[35] Torregrosa I, Montoliu C, Urios A, Elmlili N, Puchades MJ, Solis MA, et al Early biomarkers of acute kidney failure after heart angiography or heart surgery in patients with acute coronary syndrome or acute heart failure. Nefrologia. 2012;32:44–52. 10.3265/Nefrologia.pre2011.Sep.10988 22130209

[36] Kato K, Sato N, Yamamoto T, Iwasaki YK, Tanaka K, Mizuno K. Valuable markers for contrast-induced nephropathy in patients undergoing cardiac catheterization. Circ J. 2008;72:1499–1505. 10.1253/circj.cj-07-1006 18724030

[37] Ning L, Li Z, Wei D, Chen H, Yang C, Wu D, et al Urinary semaphorin 3A as an early biomarker to predict contrast-induced acute kidney injury in patients undergoing percutaneous coronary intervention. Braz J Med Biol Res. 2018;51:e6487 10.1590/1414-431X20176487 29513790 PMC5856432

[38] Liu XL, Wang ZJ, Yang Q, Yu M, Shen H, Nie B, et al Plasma neutrophil-gelatinase-associated lipocalin and cystatin C could early diagnose contrast-induced acute kidney injury in patients with renal insufficiency undergoing an elective percutaneous coronary intervention. Chin Med J (Engl). 2012;125:1051–1056. 22613530

[39] Connolly M, Kinnin M, McEneaney D, Menown I, Kurth M, Lamont J, et al Prediction of contrast induced acute kidney injury using novel biomarkers following contrast coronary angiography. QJM. 2018;111:103–110. 10.1093/qjmed/hcx201 29069419

[40] Khatami MR, Sabbagh MR, Nikravan N, Khazaeipour Z, Boroumand MA, Sadeghian S, et al The role of neutrophil-gelatinase-associated lipocalin in early diagnosis of contrast nephropathy. Indian J Nephrol. 2015;25:292–296. 10.4103/0971-4065.147370 26628795 PMC4588325

[41] Padhy M, Kaushik S, Girish MP, Mohapatra S, Shah S, Koner BC. Serum neutrophil gelatinase associated lipocalin (NGAL) and cystatin C as early predictors of contrast-induced acute kidney injury in patients undergoing percutaneous coronary intervention. Clin Chim Acta. 2014;435:48–52. 10.1016/j.cca.2014.04.016 24804935

[42] Wang M, Zhang L, Yue R, You G, Zeng R. Significance of Cystatin C for Early Diagnosis of Contrast-Induced Nephropathy in Patients Undergoing Coronary Angiography. Med Sci Monit. 2016;22:2956–2961. 10.12659/MSM.897241 27548357 PMC5004983

[43] Peng L, Wong K, Chio S, Tam K, Hun W, Tao T, et al [Diagnostic value of cystatin C in contrast-induced acute kidney injury after percutaneous coronary intervention]. Zhonghua Nei Ke Za Zhi. 2015;54:188–192. 26269438

[44] Xu Q, Wang NN, Duan SB, Liu N, Lei R, Cheng W, et al Serum cystatin c is not superior to serum creatinine for early diagnosis of contrast-induced nephropathy in patients who underwent angiography. Clin Lab Anal. 2017;31:e22096. 10.1002/jcla.22096 PMC6816834 27897324

[45] Alharazy SM, Kong N, Saidin R, Gafor AH, Maskon O, Mohd M, et al Serum neutrophil gelatinase-associated lipocalin and cystatin C are early biomarkers of contrast-induced nephropathy after coronary angiography in patients with chronic kidney disease. Angiology. 2014;65:436–442. 10.1177/0003319713483918 23580616

[46] Li S, Zheng Z, Tang X, Peng L, Luo Y, Dong R, et al Preprocedure and Postprocedure Predictive Values of Serum beta2-Microglobulin for Contrast-Induced Nephropathy in Patients Undergoing Coronary Computed Tomography Angiography: A Comparison With Creatinine-Based Parameters and Cystatin C. J Comput Assist Tomogr. 2015;39:969–974. 10.1097/RCT.0000000000000294 26248154

[47] Li S, Tang X, Peng L, Luo Y, Zhao Y, Chen L, et al A head-to-head comparison of homocysteine and cystatin C as pre-procedure predictors for contrast-induced nephropathy in patients undergoing coronary computed tomography angiography. Clin Chim Acta. 2015;444:86–91. 10.1016/j.cca.2015.02.019 25687162

[48] Nozue T, Michishita I, Mizuguchi I. Predictive value of serum cystatin C, β2-microglobulin, and urinary liver-type fatty acid-binding protein on the development of contrast-induced nephropathy. Cardiovasc Interv Ther. 2010;25:85–90. 10.1007/s12928-010-0014-3 24122467

[49] Wang L, Pu X. Predict value of monitoring changes of urinary neutrophil gelatinase-associated lipocalin and kidney injury molecule-1 after coronary angiography and percutaneous coronary intervention on early diagnosis of contrast-induced nephropathy. Zhonghua Xin Xue Guan Bing Za Zhi. 2014;42:301–304. 24924456

[50] Ling W, Zhaohui N, Ben H, Leyi G, Jianping L, Huili D, et al Urinary IL-18 and NGAL as early predictive biomarkers in contrast-induced nephropathy after coronary angiography. Nephron Clin Pract. 2008;108:c176–81. 10.1159/000117814 18287807

[51] Zhang WF, Zhang T, Ding D, Sun SQ, Wang XL, Chu SC, et al Use of Both Serum Cystatin C and Creatinine as Diagnostic Criteria for Contrast-Induced Acute Kidney Injury and Its Clinical Implications. J Am Heart Assoc. 2017;6:e004747 10.1161/JAHA.116.004747 28087509 PMC5523641

[52] Valette X, Savary B, Nowoczyn M, Daubin C, Pottier V, Terzi N, et al Accuracy of plasma neutrophil gelatinase-associated lipocalin in the early diagnosis of contrast-induced acute kidney injury in critical illness. Intensive Care Med. 2013;39:857–865. 10.1007/s00134-013-2826-y 23361630

[53] You W, Qi CL, Ye F, Huang SL, Xie DJ, Wu ZM, et al The value of urinary neutrophil gelatinase-associated lipocalin for early diagnosis of contrast-induced nephropathy. Zhonghua Xin Xue Guan Bing Za Zhi. 2016;44:1024–1029. 10.3760/cma.j.issn.0253-3758.2016.12.007 28056233

[54] Herget-Rosenthal S, Marggraf G, Husing J, Goring F, Pietruck F, Janssen O, et al Early detection of acute renal failure by serum cystatin C. Kidney Int. 2004;66:1115–1122. 10.1111/j.1523-1755.2004.00861.x 15327406

[55] Dharnidharka VR, Kwon C, Stevens G. Serum cystatin C is superior to serum creatinine as a marker of kidney function: a meta-analysis. Am J Kidney Dis. 2002;40:221–226. 10.1053/ajkd.2002.34487 12148093

[56] Bachorzewska-Gajewska H, Malyszko J, Sitniewska E, Malyszko JS, Pawlak K, Mysliwiec M, et al Could neutrophil-gelatinase-associated lipocalin and cystatin C predict the development of contrast-induced nephropathy after percutaneous coronary interventions in patients with stable angina and normal serum creatinine values? Kidney Blood Press Res. 2007;30:408–415. 10.1159/000109102 17901710

[57] Charlton JR, Portilla D, Okusa MD. A basic science view of acute kidney injury biomarkers. Nephrol Dial Transplant. 2014;29:1301–1311. 10.1093/ndt/gft510 24385545 PMC4081632

[58] Singer E, Marko L, Paragas N, Barasch J, Dragun D, Muller DN, et al Neutrophil gelatinase-associated lipocalin: pathophysiology and clinical applications. Acta Physiol (Oxf). 2013;207:663–672. 23375078 10.1111/apha.12054 PMC3979296

[59] Bachorzewska-Gajewska H, Poniatowski B, Dobrzycki S. NGAL (neutrophil gelatinase-associated lipocalin) and L-FABP after percutaneous coronary interventions due to unstable angina in patients with normal serum creatinine. Adv Med Sci. 2009;54:221–224. 10.2478/v10039-009-0036-1 19875355

[60] Malyszko J, Bachorzewska-Gajewska H, Poniatowski B, Malyszko JS, Dobrzycki S. Urinary and serum biomarkers after cardiac catheterization in diabetic patients with stable angina and without severe chronic kidney disease. Ren Fail. 2009;31:910–919. 10.3109/08860220903216113 20030526

[61] Morcos SK, Thomsen HS, Webb JA. Contrast-media-induced nephrotoxicity: a consensus report. Contrast Media Safety Committee, European Society of Urogenital Radiology (ESUR). Eur Radiol. 1999;9:1602–1613. 10.1007/s003300050894 10525875

[62] Stacul F, van der Molen AJ, Reimer P, Webb JA, Thomsen HS, Morcos SK, et al Contrast induced nephropathy: updated ESUR Contrast Media Safety Committee guidelines. Eur Radiol. 2011;21:2527–2541. 10.1007/s00330-011-2225-0 21866433

[63] Mehran R, Aymong ED, Nikolsky E, Lasic Z, Iakovou I, Fahy M, et al A simple risk score for prediction of contrast-induced nephropathy after percutaneous coronary intervention: development and initial validation. J Am Coll Cardiol. 2004;44:1393–1399. 10.1016/j.jacc.2004.06.068 15464318

[64] McCullough PA, Choi JP, Feghali GA, Schussler JM, Stoler RM, Vallabahn RC, et al Contrast-Induced Acute Kidney Injury. J Am Coll Cardiol. 2016;68:1465–1473. 10.1016/j.jacc.2016.05.099 27659469

[65] Steubl D, Inker LA. How best to estimate glomerular filtration rate? Novel filtration markers and their application. Curr Opin Nephrol Hypertens. 2018;27:398–405. 10.1097/MNH.0000000000000444 30063487

[66] Murray PT, Mehta RL, Shaw A, Ronco C, Endre Z, Kellum JA, et al Potential use of biomarkers in acute kidney injury: report and summary of recommendations from the 10th Acute Dialysis Quality Initiative consensus conference. Kidney Int. 2014;85:513–521. 10.1038/ki.2013.374 24107851 PMC4198530

[67] Stacul F, van der Molen AJ, Reimer P, Webb JA, Thomsen HS, Morcos SK, et al Contrast induced nephropathy: updated ESUR Contrast Media Safety Committee guidelines. Eur Radiol. 2011;21:2527–2541. 10.1007/s00330-011-2225-0 21866433

[68] Mehta RL, Kellum JA, Shah SV, Molitoris BA, Ronco C, Warnock DG, et al Acute Kidney Injury Network: report of an initiative to improve outcomes in acute kidney injury. Crit Care. 2007;11:R31 10.1186/cc5713 17331245 PMC2206446

[69] Krzeminska E, Wyczalkowska-Tomasik A, Korytowska N, Paczek L. Comparison of Two Methods for Determination of NGAL Levels in Urine: ELISA and CMIA. J Clin Lab Anal. 2016;30:956–960. 10.1002/jcla.21962 27075972 PMC6806694

[70] Itenov TS, Bangert K, Christensen PH, Jensen JU, Bestle MH. Serum and plasma neutrophil gelatinase associated lipocalin (NGAL) levels are not equivalent in patients admitted to intensive care. J Clin Lab Anal. 2014;28:163–167. 10.1002/jcla.21662 24395189 PMC6807606

[71] Li J, Dunn W, Breaud A, Elliott D, Sokoll LJ, Clarke W. Analytical performance of 4 automated assays for measurement of cystatin C. Clin Chem. 2010;56:1336–1339. 10.1373/clinchem.2009.141531 20562350

[72] Hansson LO, Grubb A, Liden A, Flodin M, Berggren A, Delanghe J, et al Performance evaluation of a turbidimetric cystatin C assay on different high-throughput platforms. Scand J Clin Lab Invest. 2010;70:347–353. 10.3109/00365513.2010.491124 20545461

[73] Delanaye P, Pieroni L, Abshoff C, Lutteri L, Chapelle JP, Krzesinski JM, et al Analytical study of three cystatin C assays and their impact on cystatin C-based GFR-prediction equations. Clin Chim Acta. 2008;398:118–124. 10.1016/j.cca.2008.09.001 18805407

[74] Flodin M, Hansson LO, Larsson A. Variations in assay protocol for the Dako cystatin C method may change patient results by 50% without changing the results for controls. Clin Chem Lab Med. 2006;44:1481–1485. 10.1515/CCLM.2006.271 17163826

