# Supplementary material for: Predictive value of cystatin C and neutrophil gelatinase-associated lipocalin in contrast-induced nephropathy: A meta-analysis
Source: PLoS One. 2020 Apr 2;15(4):e0230934. doi: 10.1371/journal.pone.0230934 (PMC7117687; doi:10.1371/journal.pone.0230934)
Supplement: S1 Table — (DOCX) [file pone.0230934.s002.docx]

**S1 Table** **Meta-regression analyses for potential sources of heterogeneity from each group.**

| **Covariate** | **No. of studies** | **Sensitivity%(95%CI)** | **P_sen_ value** | **Specificity%(95%CI)** | **P_spe_ value** | **P value (Joint model)** |
| --- | --- | --- | --- | --- | --- | --- |
| **blood NGAL** |  |  |  |  |  |  |
| **CIN definition threshold** | |  |  |  |  |  |
| 0.3mg/dl | 3 | 72(43-100) | 0.18 | 76(55-97) | 0.97 | 0.35 |
| 0.5mg/dl | 6 | 91(81-100) |  | 82(69-95) |  |  |
| **CIN definition time** |  |  |  |  |  |  |
| within 48h | 4 | 69(47-92) | 0.02 | 81(65-97) | 0.66 | **0.04** |
| over 48h or NR | 5 | 93(86-100) |  | 79(64-95) |  |  |
| **risk** |  |  |  |  |  |  |
| CKD | 5 | 79(59-98) | 0.22 | 83(70-96) | 0.90 | 0.26 |
| others | 4 | 92(81-100) |  | 77(58-95) |  |  |
| **Assay** |  |  |  |  |  |  |
| biochips | 1 | 76(21-100) | 0.94 | 96(90-100) | 0.06 | **0.02** |
| other assays | 8 | 88(76-100) |  | 75(65-86) |  |  |
| **sample source** |  |  |  |  |  |  |
| plasma | 3 | 64(42-87) | 0.01 | 84(68-100) | 0.89 | **0.01** |
| serum | 6 | 91(84-99) |  | 79(64-93) |  |  |
| **urine NGAL** |  |  |  |  |  |  |
| **CIN definition threshold** | |  |  |  |  |  |
| 0.3mg/dl | 6 | 80(69-90) | 0.95 | 82(70-94) | 0.52 | 0.27 |
| 0.5mg/dl | 4 | 92(85-99) |  | 75(64-87) |  |  |
| **CIN definition time** |  |  |  |  |  |  |
| NR | 3 | 92(82-100) | 0.71 | 90(83-97) | 0.86 | **0.02** |
| within 48/72h | 7 | 83(73-93) |  | 71(62-80) |  |  |
| **risk** |  |  |  |  |  |  |
| CKD | 3 | 91(80-100) | 0.76 | 78(62-94) | 0.29 | 0.56 |
| others | 7 | 83(72-95) |  | 78(68-88) |  |  |
| **Assay** |  |  |  |  |  |  |
| Architect platform | 2 | 70(54-87) | <0.0001 | 67(46-88) | 0.07 | 0.11 |
| others | 8 | 89(84-95) |  | 80(72-89) |  |  |
| **Location** |  |  |  |  |  |  |
| Brazil | 1 | 59(23-95) | 0.03 | 81(57-100) | 0.92 | **0.02** |
| others | 9 | 89(82-95) |  | 78(69-87) |  |  |
| **serum cystatin C (after ommiting the most particular study, Suhua Li a)** | | | | |  |  |
| **CIN definition threshold** | |  |  |  |  |  |
| 0.3mg/dl | 5 | 83(63-100) | 0.90 | 83(71-94) | 0.10 | 0.98 |
| 0.5mg/dl | 12 | 85(72-97) |  | 83(76-90) |  |  |
| **CIN definition time** |  |  |  |  |  |  |
| within 48h | 12 | 85(72-97) | 0.90 | 86(81-92) | 0.63 | 0.15 |
| over 48h or NR | 5 | 83(63-100) |  | 73(59-86) |  |  |
| **Assay** |  |  |  |  |  |  |
| laboratory | 1 | 84(44-100) | 0.57 | 98(95-100) | 0.04 | **0.02** |
| others | 16 | 84(73-95) |  | 81(75-86) |  |  |
| **risk** |  |  |  |  |  |  |
| CKD | 3 | 80(51-100) | 0.74 | 88(78-98) | 0.47 | 0.60 |
| others | 14 | 85(74-96) |  | 82(75-88) |  |  |

Boldface type means statistical significance (P<0.05). NGAL, neutrophil gelatinase-associated lipocalin; 95%CI, 95% confidence interval; CIN, contrast-induced nephropathy; laboratory, primary study didn’t mention the specific assay but measuring cystatin C in hospital laboratory
