# Supplementary material for: Predictive value of cystatin C and neutrophil gelatinase-associated lipocalin in contrast-induced nephropathy: A meta-analysis
Source: PLoS One. 2020 Apr 2;15(4):e0230934. doi: 10.1371/journal.pone.0230934 (PMC7117687; doi:10.1371/journal.pone.0230934)
Supplement: Attachment — Submitted filename: Response to reviewers.docx [file pone.0230934.s003.docx]

Dear Editors,

Firstly, we would like to thank you for your and reviewers’ constructive comments on our manuscript (No. PONE-D-19-34745) entitled “Predictive value of cystatin C and neutrophil gelatinase-associated lipocalin in contrast-induced nephropathy: a systematic review and meta-analysis”. These comments are all valuable and helpful for improving our article. According to the academic editor’s and reviewers’ comments, we have made an extensive modification on the revised manuscript and all changes within the document were also highlighted by using red-colored text. Point-by-point responses are listed below the letter.

**Answers to academic editor:**

Please ensure that your manuscript meets PLOS ONE’s style requirements, including those for file naming.

**Answer:** Thanks for your nice suggestions. We have modulated the format of the manuscript according to the PLOS ONE’s guidelines and tried to meet all requirements. We hope this version would be suitable.

To comply with the items on the PRISMA checklist, please your abstract using subheadings.

**Answer:** We are so sorry for our carelessness. Abstract has been revised as a structured one. Besides, the PRISMA checklist is uploaded as supporting information.

We note that you have reported significance probabilities of 0 in places. Since p=0 is not strictly possible, please correct this to a more appropriate limit, eg 'p<0.0001'.

**Answer:** Thanks. We have corrected mistakes based on your suggestions.

Thank you for stating the following financial disclosure: the funders had no role in study design, data collection and analysis, decision to publish, or preparation of the manuscript.'

a. Please provide an amended Funding Statement that declares *all* the funding or sources of support received during this specific study (whether external or internal to your organization) as detailed online in our guide for authors at <http://journals.plos.org/plosone/s/submit-now>
b. Please state what role the funders took in the study.  If any authors received a salary from any of your funders, please state which authors and which funder. If the funders had no role, please state: "The funders had no role in study design, data collection and analysis, decision to publish, or preparation of the manuscript."

c. Please include your amended statements within your cover letter; we will change the online submission form on your behalf.

**Answer:** Thank you very much for your nice suggestions. We have amended the financial statement according to the guide and included those contents in cover letter.

Please include a copy of Table 5 which you refer to in your text on page 22.

**Answer:** We are sorry for our neglection that we have renamed Table 5 as S1 Table. We have revised the Table’s name mentioned in the text.

Please include captions for your Supporting Information files at the end of your manuscript, and update any in-text citations to match accordingly.

**Answer:** We are sorry for our neglect. The captions of Supporting Information has been included at the end of the manuscript. Also, all citations have been updated and modulated based on the recommended style.

**Answers to reviewers:**

**Reviewer #1**

Page 3 line 58, please select decisions or interventions, not both.

**Answer:** We are really sorry for our careless mistake. It has been corrected as “clinical interventions” only.

Page 4 line 67. I suggest to add: "and catabolized by the tubular cells".

**Answer:** Thanks for your nice suggestion. We have added the above information and the sentence has been revised as “Cystatin C is a 13-kDa cysteine proteinase inhibitor produced by nucleated cells and can be freely filtered by glomeruli, then reabsorbed and catabolized by the tubular cells”.

Page 4 lines 81 and 82. Systematic instead systemic.

**Answer:** We are really sorry for the spelling mistakes. We have corrected both words on your reminding.

Page 5 line 97, the same in the title of Table 1.

**Answer:** We are really sorry for the spelling mistakes. We have corrected the word on your reminding.

Page 6 line 120, pleas paid attention to the wording.

**Answer:** We are sorry for the ambiguous expression here. The sentence has been revised as “The extracted data included study characteristics (first author, publication year, study region, study design, and CIN/contrast-induced AKI definition), patient characteristics (number of patients, age, sex distribution, baseline renal function and settings) and index test characteristics (the detection method of index, evaluation time, sample source, and cut-off value)”.

Page 6 line 122, in "patient characteristic" comorbidities are not mentioned being relevant in CIN. However, were included in the analysis. Please correct this inconsistency.

**Answer:** Thanks for your reminding. We have corrected the mistake on your suggestions.

Page 12 Table 3. Paid attention to table design. When name and last name of first author were included the line is double but stays single in the remaining columns.

**Answer:** Thanks for your careful scrutiny. We have modulated the expression of the author name and further organized the format of tables. The line of every column stays single now.

Page 19 line 307, I found confusing the sentence "After kidney injury, the rise in serum cystatin C levels can be used to detect even a minor glomerular filtration rate (GFR) reduction". Please clarify.

**Answer:** We are sorry for the ambiguous and imprecise expression of this. The sentence has been revised as “After kidney injury, the rise of cystatin C is much earlier and superior to sCr in detecting reduced glomerular filtration rate (GFR)”

Page 22 line 362, ESUR definition and AKIN definition are mixed. Relative increase of SCr and time limit are crossed.

**Answer:** We appreciate your constructive comment. We have corrected the mistake and tried to organize the sentence clearer.

There are many references there in plain text. Please check for abbreviations.

**Answer:** Thanks for your kind suggestion. We have formatted all the references and checked the abbreviations of Journals according to [National Center for Biotechnology Information (NCBI) databases](http://www.ncbi.nlm.nih.gov/nlmcatalog/journals).

**Reviewer #2:**

The systematic review and meta-analysis performed by Yi et al addresses an important and practical issue, i.e., the diagnostic performance of renal biomarkers, NGAL AND CYSTATIN C, in contrast induced nephropathy. A careful and detailed statistical analysis was performed pertaining the appropriate literature published until November 2019. They concluded that both NGAL and especially Cystatin C, may serve as early diagnostic indicators of CIN.

**Answer:** Thanks for your positive comments.
